# Phylogeny and Mycotoxin Profile of Pathogenic *Fusarium* Species Isolated from Sudden Decline Syndrome and Leaf Wilt Symptoms on Date Palms (*Phoenix dactylifera*) in Tunisia

**DOI:** 10.3390/toxins13070463

**Published:** 2021-06-30

**Authors:** Amal Rabaaoui, Chiara Dall’Asta, Laura Righetti, Antonia Susca, Antonio Francesco Logrieco, Ahmed Namsi, Radhouane Gdoura, Stefaan P. O. Werbrouck, Antonio Moretti, Mario Masiello

**Affiliations:** 1 Department of Plants and Crops, Faculty of Bioscience Engineering, Ghent University, 9000 Ghent, Belgium; rabaaouiamal1@gmail.com (A.R.); Stefaan.Werbrouck@ugent.be (S.P.O.W.); 2 Laboratory of Toxicology-Microbiology and Environmental Health, Department of Biology, University Sfax, Sfax 3000, Tunisia; gdoura.radhouane@gmail.com; 3 Department of Food and Drug, University of Parma, Area delle Scienze 27/A, 43124 Parma, Italy; chiara.dallasta@unipr.it (C.D.); laura.righetti@unipr.it (L.R.); 4 National Research Council of Italy, Institute of Sciences of Food Production, CNR-ISPA, Via Amendola 122/O, 70126 Bari, Italy; antonella.susca@ispa.cnr.it (A.S.); antonio.logrieco@ispa.cnr.it (A.F.L.); mario.masiello@ispa.cnr.it (M.M.); 5 Laboratoire de Phytopathologie, Centre Régional de Recherches en Agriculture Oasienne, Degache 2260, Tunisia; ahmed_en@yahoo.fr

**Keywords:** *Fusarium prolferatum*, *Fusarium brachygibbosum*, *Fusarium solani*, Fusarium Equiseti Incarnatum species complex, fumonisins

## Abstract

In 2017–2018, extensive symptoms of sudden decline and fruit rot were observed on date palms in southern Tunisia. Samples of diseased plants were randomly collected in six localities. Based on morphological identification, *Fusarium* was the most frequent fungal genus detected. A sequencing of translation elongation factor, calmodulin, and second largest subunit of RNA polymerase II genes was used to identify 63 representative *Fusarium* strains at species level and investigate their phylogenetic relationships. The main species detected was *Fusarium proliferatum*, and at a much lesser extent, *Fusarium brachygibbosum*, *Fusarium caatingaense*, *Fusarium clavum*, *Fusarium incarnatum,* and *Fusarium solani*. Pathogenicity on the *Deglet Nour* variety plantlets and the capability to produce mycotoxins were also assessed. All *Fusarium* species were pathogenic complying Koch’s postulates. *Fusarium proliferatum* strains produced mainly fumonisins (FBs), beauvericin (BEA), and, to a lesser extent, enniatins (ENNs) and moniliformin (MON). All *F. brachygibbosum* strains produced low levels of BEA, diacetoxyscirpenol, and neosolaniol; two strains produced also T-2 toxin, and a single strain produced HT-2 toxin. *Fusarium caatingaense*, *F. clavum*, *F. incarnatum* produced only BEA. *Fusarium solani* strains produced MON, BEA, and ENNs. This work reports for the first time a comprehensive multidisciplinary study of *Fusarium* species on date palms, concerning both phytopathological and food safety issues.

## 1. Introduction 

Date palm (*Phoenix dactylifera* L.) crop covers an area of about 1.1 million hectares worldwide, with a production of approximately 8,500,000 tons [1]. The main date-producing regions are Asia and Africa, with 56% and 43% of the world’s harvest, respectively. In Tunisia, the total production of dates has reached the highest level ever in 2018, with about 305 thousand tons of fruit produced, half of which destined to the export [1]. Therefore, in Tunisia, the date palm occupies a strategic place in the socio-economic stability of the oasis agro-system in desert regions and provides the main financial resource of the oasis. Indeed, an analysis on the competitive advantage of the date industry in the all Mediterranean area countries and Iran showed that Tunisia is the main supplier of dates to the EU, with about 10% of the Tunisian population depending on this crop [2].

In all cultivated areas worldwide, date palms, under suitable climatic conditions, are susceptible to various fungal pathogens, especially to *Fusarium* species [3,4]. In particular, the most severe pathogen is *F. oxysporum*, *F*. *albedinis* that causes Fusarium wilt of date palm trees, the Bayoud disease [4,5] and has been responsible of the death of more than 15,000,000 trees in Morocco and Algeria in the past [6]. Currently, strict phytosanitary rules are applied at the borders of date-palm countries that remain free of Bayoud, being this disease mostly spread in Northern Africa [5]. However, other *Fusarium* species have also been reported as causal agents of sudden the decline syndrome of date palms. *F. proliferatum* in particular is reported to be highly pathogenic in many countries where date palms are grown. In both Saudi Arabia and Spain, several authors have isolated this species from symptomatic young plants, leaves, or roots [3,7,8], or rotten fruit, as in Israel and Iran [9,10]. Another *Fusarium* species that has been significantly reported as a serious pathogen of date palms is *F. solani*, isolated from date palm wilted leaves and roots, in several countries such as Iran, Egypt, Oman, Pakistan, Saudi Arabia, United Arab Emirates, and Qatar [3,11,12,13,14,15,16]. In addition, a single strain of *F. brachygibbosum* and *F. verticillioides*, isolated from symptomatic date palm roots, were reported by Saleh et al. [3], while Abbas et al. [17] reported the identification of *F. equiseti* isolated from date palm roots in Iraq. Nishad and Ahmed [16] isolated the same species from wilted leaves in Qatar. Finally, the latter authors reported also a significant occurrence of *F. brachygibbosum* on wilted leaves and roots of date palms in Qatar [16].

A wide biodiversity characterizes the *Fusarium* genus, which species occur on several different crops and geographical areas, and their frequency is related to both climatic conditions and cropping practices [5,18]. *Fusarium* species cannot only be devastating plant pathogens or secondary invaders, colonizing plants throughout the whole growth cycle, but they can also produce and accumulate several secondary toxic metabolites, the mycotoxins, in plant tissues. Therefore, they also pose a serious risk to food safety due to the consumption of mycotoxin-contaminated crop products [19]. *Fusarium* species can produce mycotoxins under the influence of environmental factors, such as moisture, temperature, carbon dioxide, oxygen, substrate composition, and agronomic factors such as pesticides used, and plant variety susceptibility [20,21]. Moreover, *Fusarium* species are characterized by a wide inter- and intraspecific genetic diversity that can explain the dramatic variability of their mycotoxin profile that often is species-specific. The range of mycotoxins produced by this fungal genus is wide and includes trichothecenes, potent inhibitors of protein synthesis, zearalenone (ZEA), related to estrogenic disorders in human and animals, the carcinogenic fumonisins (FBs), classified by the International Agency of Cancer Research as group 2B [22,23], and other mycotoxins of lower concern for their toxicity such as moniliformin (MON), beauvericin (BEA), and enniatins (ENNs), but often reported at high amounts in crop products [19]. All mycotoxins cited above can be produced by a single or multiple *Fusarium* species reported to occur on date palm plants worldwide. Therefore, establishing the mycotoxin profile for the species occurring on date palm plants is an important step for a correct risk assessment related to *Fusarium*-mycotoxins contamination of date palm. 

**Figure 1:**
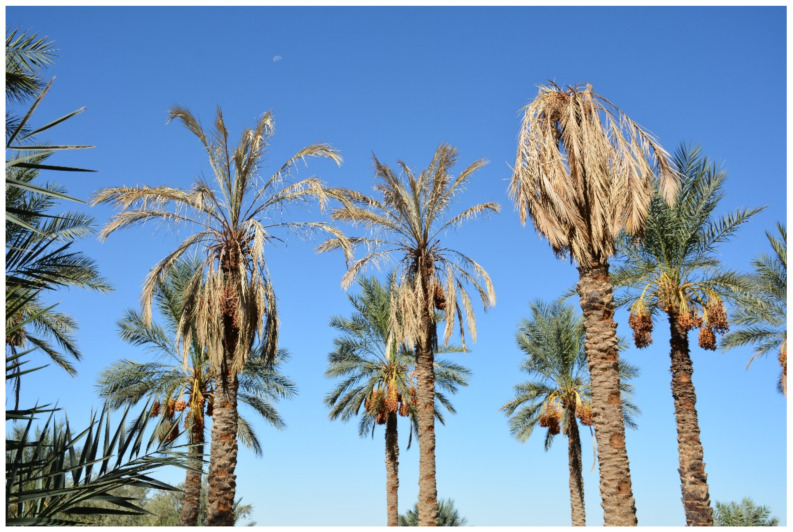
A group of date palm trees showing symptoms of sudden decline syndrome.

Due to the appearance of different disease symptoms on date palm leaves in South Tunisia, in 2017 (Figure 1), mainly wilt and dieback, a collection of date palm plant samples, from symptomatic roots and leaflets, was collected in both 2017 and 2018. The isolation from the samples led to the morphological identification of the fungal strains as mainly belonging to the *Fusarium* genus.

The aims of this study were (i) identifying the *Fusarium* species isolated from symptomatic date palm plants; (ii) assessing their pathogenicity on date palm plantlets of the most common Tunisian variety Deglet Nour; (iii) establishing phylogenetic relationships of the *Fusarium* species isolated; (iv) describing the mycotoxin profile of the main *Fusarium* species identified on date palm plants in Tunisia.

## 2. Results

### 2.1. Phylogenetic Analyses

The evolutionary history of 63 *Fusarium* strains was studied at the genetic level by amplifying fragments of three different genes: calmodulin (*CAL1*), second largest subunit of RNA polymerase II (*RPB2*), and translation elongation factor (*TEF1*). 

**Figure 2:**
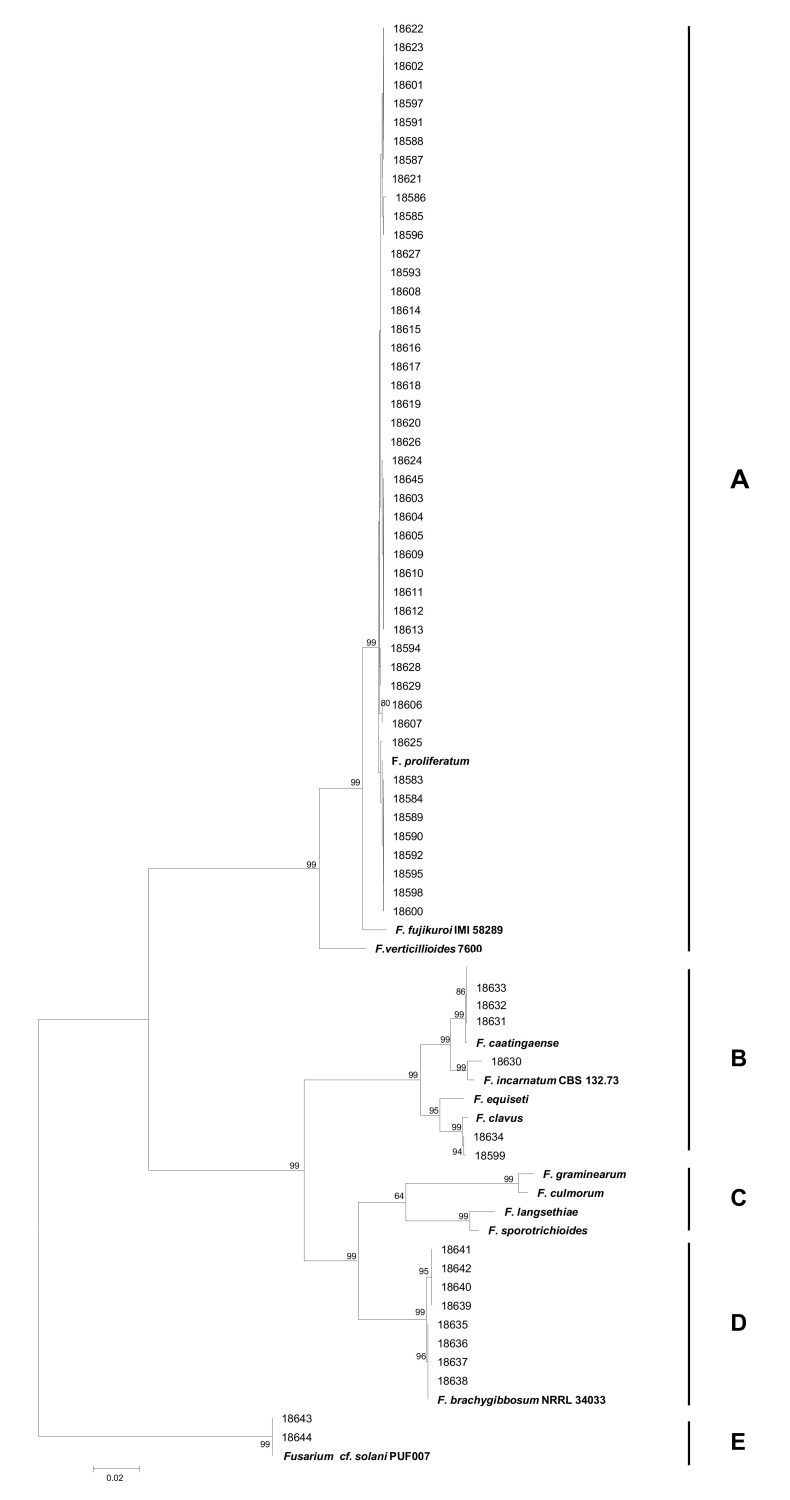
Phylogenetic tree generated by Maximum Likelihood method (bootstrap 1000 replicates) from combined DNA sequences of *CAL1*, *RPB2,* and *TEF1* gene fragments of 63 *Fusarium* strains.

To further solve the identity of the *Fusarium* strains, the three gene sequences *CAL1*, RPB2, and *TEF1* were concatenated and analyzed simultaneously with species reference strains (Table 1). In particular, common fragments around 590 bp (640 bp in *F. solani* strains) 870 bp, and 550 bp, respectively, were considered. A phylogenetic analysis of the concatenated sequences of about 2000 nt was carried out. The phylogenetic tree (Figure 2), generated by the Mega7 software using the Maximum Likelihood method, allowed us to define five well-separated clades (supported by high bootstrap values of 99), corresponding to *Fusarium fujikuroi* species complex (FFSC, A), *Fusarium incarnatum equiseti* species complex (FIESC, B), *Fusarium sambucinum* species complex (FSAMSC, C), *F. brachygibossum* (D), and *Fusarium solani* species complex (FSSC, E), as determined using reference strains downloaded from NCBI (Table 1, Figure 2).

In clade A, where the reference strains of the main species belonging to FFSC, 47 *Fusarium* strains clustered with *F. proliferatum* reference sequences. In particular, eight *Fusarium* strains (ITEM18583, ITEM18584, ITEM18589, ITEM18590, ITEM18592, ITEM18595, ITEM18598, ITEM18600) showed a higher homology than other *F. proliferatum* field strains with *F. proliferatum* reference strain.

A high variability was observed in clade B in which six *Fusarium* strains grouped with FIESC references included in the analyses, *F. caatingaense*, *F. incarnatum*, *F. equiseti*, and *F. clavum*. In particular, the strains ITEM18631, ITEM18632, ITEM18633, with 100% of homology among them, showed a high homology with *F. caatingaense* reference species. *Fusarium* strain ITEM18630 was very similar to *F. incrnatum* and two strains (ITEM18634, ITEM18599) were identified as *F. clavum*. In clade C, only reference sequences of *F. graminearum*, *F. culmorum*, *F. langhsethiae,* and *F. sporotrichioides* were included, since no *Fusarium* species belonging to this species complex were isolated from date palm plants. 

In clade D, eight *Fusarium* field strains clustered with the *F. brachygibbosum* reference strain. In particular, *Fusarium* ITEM18635, ITEM18636, ITEM18637, ITEM18638 strains showed 100% homology with reference sequences. The strains ITEM18639, ITEM18640, ITEM18641, and ITEM18642 showed 100% of homology among them and were very close to the other group (Figure 2).

Finally, two *Fusarium* strains (ITEM18643, ITEM18644), showing 100% of homology with *F. solani* PUF007 strain, were grouped together in a well-defined clade (E).

### 2.2. Pathogenicity Assay

**Figure 3:**
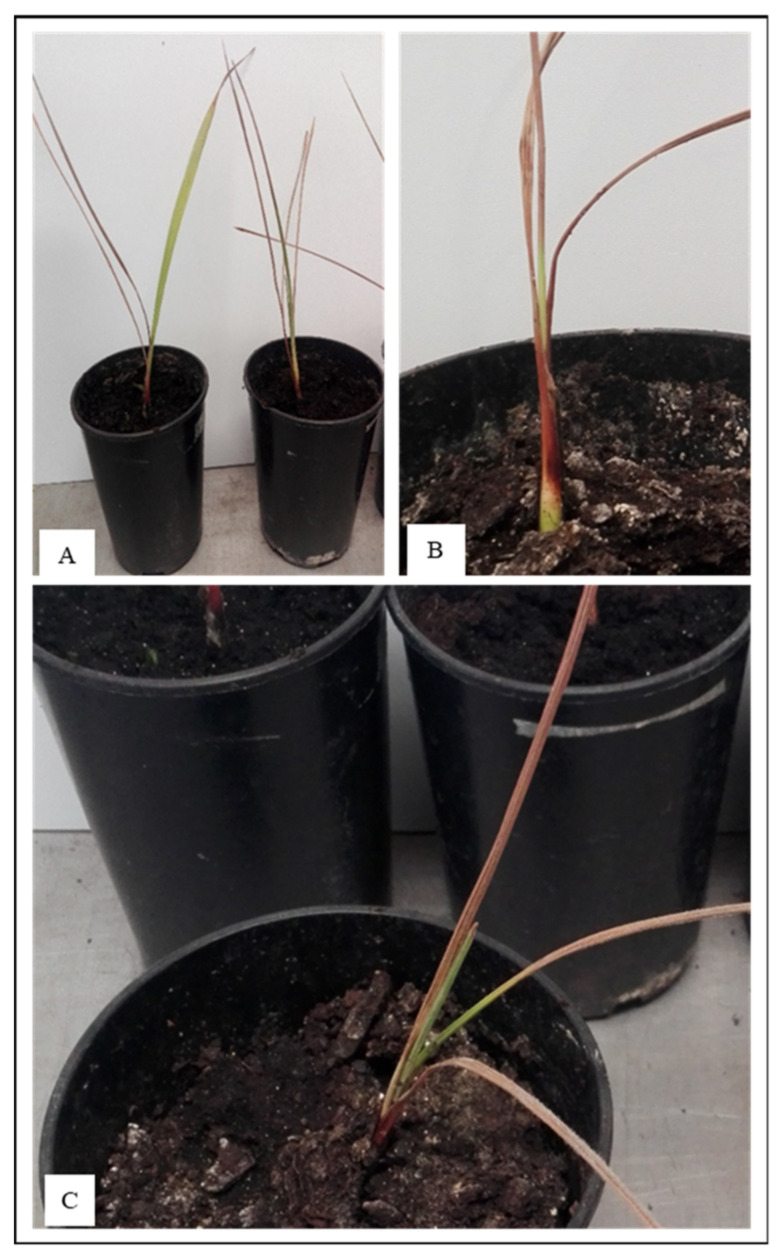
Symptoms of *Fusarium* infections on date palm plantlets *cv* Deglet Nour, showing general whitening and dryness of stem and leaf tissues and sudden decline, 20 days after inoculation with *Fusarium proliferatum* ITEM 18584 strain (**A**), *Fusarium brachygibbosum* ITEM 18635 strain (**B**) and *Fusarium caatingaense* ITEM18631 strain (**C**).

Data on the pathogenicity are summarized in Table S1. Following inoculation with *F. solani,* and FIESC members, *F. caatingaense*, *F. clavum*, and *F. incarnatum*, the plantlets developed typical symptoms of sudden decline, such as yellowing leaves from the upper part to the lower leaflets, followed by total whitening that appeared at 20 days post inoculation (dpi) (Figure 3). Furthermore, the stems showed distinct brown or dark spots (Figure 3). In plants inoculated with *F. proliferatum,* the infection led to dryness and wilting leaflets after 20 dpi, with typical symptoms of leaf wilt (Figure 3). Finally, the infection by using *F. brachygibbosum* developed mild leaf yellowing symptoms (Figure 3). 

All strains tested were re-isolated and were identical to those isolated from leaflets infected in the open field, confirming Koch’s postulates. Control plants lacked symptoms.

The DSI was calculated for all strains tested: *F. proliferatum* DSI varied from 38.9 to 83.3 among the 37 strains tested; the DSI of seven strains belonging to *F. brachygibbosum* varied from 41.7 to 80.6; DSI of strains belonging to FIESC varied from to 58.3 to 88.9; finally, the two strains of *F. solani* had a DSI of 72.2 and 80.6.

**Figure 4:**
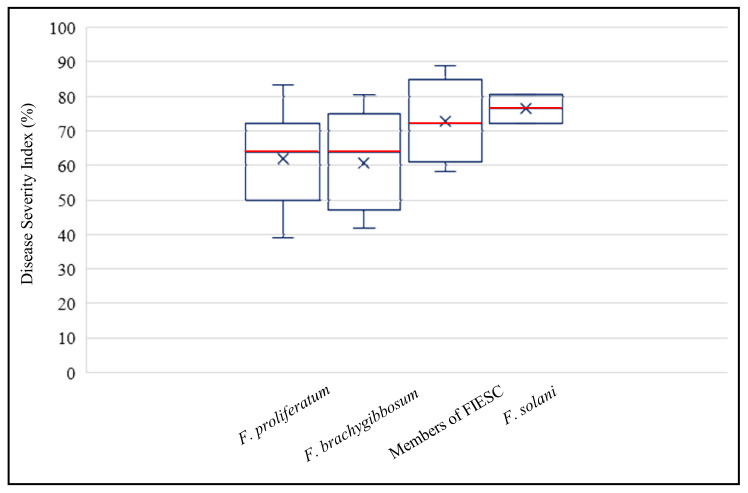
Box plots (median, red line, upper, and lower 25th and 75th percentiles) of Disease Severity Index calculated for the different *Fusarium* species inoculated on date palm plantlets *cv* Deglet Nour.

The analysis of variance revealed that there was not significant difference of pathogenicity rates among the *Fusarium* species and the distributions of DSI was not separated among the *Fusarium* species, as indicated by the box plots in Figure 4.

In particular, for *F. proliferatum*, the species with biggest number of strains tested, the data were analyzed by comparing strains grouped based on the locality of origin. No significant differences in pathogenicity were recorded from groups of different regions (Table S1). 

### 2.3. Mycotoxin Production

Data of mycotoxin production in vitro are reported in Table 2. 

*Fusarium proliferatum*: all strains were able to produce at least one mycotoxin. With regard to FBs, six strains did not produce FBs, while 41 strains produced FB_1_ in a range from 0.8 to 11809.8 mg kg^−1^ and FB_2_ in a range from 0.3 to 808.9 mg kg^−1^. Moreover, 37 strains produced FB_3_ in a range from 0.6 to 803.1 mg kg^−1^ Therefore, among the 47 *F. proliferatum* strains analyzed, four strains could produce FB1 and FB2 but were not able to produce FB3. 

All strains were able to produce BEA in a range from 0.1 to 437.4 mg kg^−1^.

Only eight strains were able to produce MON, with values ranging between 1 and 41.4 mg kg^−1^. 

Only a single strain was able to produce all ENNs: ENNA1 (112.4 mg kg^−1^) ENNB (48.6 mg kg^−1^), ENNB1 (33.7 mg kg^−1^), and ENNH (51.1 mg kg^−1^). A very low amount of ENNB was detected in two strains, with values of 0.1 and 0.06 mg kg^−1^, respectively. Only a single strain produced also ENNH, but at a low amount (1.1 mg kg^−1^).

*Fusarium brachygibbosum*: all strains produced low amount of BEA with values ranging between 0.1 and 1.9 mg kg^−1^. Only a single strain was able to produce a low amount of ENNB (0.02 mg kg^−1^) and ENNH (1.9 mg kg^−1^). Traces of DAS and NEO were found in all the strains, while a single strain was able to produce also HT2 toxin (18.7 mg kg^−1^). Also for T2 toxin production, a single strain synthetized the mycotoxin with a value of 1.2 mg kg^−1^.

FIESC: members of this complex were evaluated for their capability to produce MON, BEA, ENNs, and trichothecenes. All five strains analyzed were able to produce only BEA. In particular, with the exception of a strain that produced BEA at a high level (423.1 mg kg^−1^), all strains produced low amounts of BEA, with values ranging between 0.3 and 3.2 mg kg^−1^. 

*Fusarium solani*: The two strains analyzed produced MON (50.1 and 62.2 mg kg^−1^), BEA (21.6 and 25.2 mg kg^−1^), ENNA1 (82.5 and 95.1 mg kg^−1^), and ENNH (285 and 224.1 mg kg^−1^). Only a single strain produced ENNB (8.2 mg kg^−1^).

## 3. Discussion

### 3.1. Species Identification

In this study, we report for the first time the occurrence of *Fusarium* species on date palm plants in Tunisia that show evident symptoms of disease. The prevalence was extensive in several places and the incidence was high. Among the *Fusarium* species, the most harmful pathogen *F. oxysporum* f. sp. *albedinis*, the causal agent of the destructive Bayoud Disease [5], was not detected, although this pathogen is currently found in Algeria, Morocco, and Mauritania. For this reason, *F. oxysporum* f. sp. *albedinis* is subject to strict phytosanitary regulations at the borders of neighboring countries that are free of Bayoud’s disease [32]. Based on phylogenetic analysis of three loci, we identified in this survey *F. proliferatum*, a member of FFSC, as the dominant species. To a lesser extent, also *F. brachygibbosum*, member of FSAMSC, and three members of FIESC, *F. incarnatum, F. clavum,* and *F. caatingaense*, were isolated and identified. Finally, rarely, also strains of *F. solani*, a species member of the FSSC, were identified. Our data confirm previous worldwide reports on the occurrence and pathogenicity of *F. proliferatum* on date palms [3,7,9,10,15]. Some of these reports attribute only a low or occasional frequency to this species [7,10,15], while in Israel [9] and Saudi Arabia [3] *F. proliferatum* occurred at high incidence in symptomatic plants in various localities. On the other hand, the occurrence of *F. proliferatum* has also been reported here for the first time also for the whole of northern Africa, while the previous reports were from Asia [3,7,9,10,15] and Europe [8]. 

### 3.2. Pathogenicity Test

In our pathogenicity test, carried out on the the Deglet Nour variety, which represents 80% of Tunisian production, *F. proliferatum* proved to be the least pathogenic of the *Fusarium* species tested, namely, moderately pathogenic, according to the scale that we used. However, the group of *F. proliferatum* strains isolated from Chabbat and Mides proved to have a similar mean level of pathogenicity as *F. brachygibbosum*, *F. solani,* and *Fusarium* species members of FIESC. Therefore, also within this species, strains with high pathogenic potential occurred which could provide a genetic source for increasing their pathogenicity on date palms. This confirms previous reports from other geographical areas on the high pathogenicity of *F. proliferatum*, assessed by different pathogenic bioassays [3,7,8,10]. On the other hand, although Alwashi et al. [15] isolated *F. proliferatum* at high frequency from date palm roots affected by Sudden Disease Syndrome in UAE, they reported that this species was not pathogenic on date palm plantlets. Further studies should be carried out to better assess the effective role played by *F. proliferatum* on the fungal diseases affecting date palm. 

Pathogenicity was tested also for other species identified in this survey that occurred to a lesser extent. The pathogenicity of the two strains of *F. solani* was the highest with a DSI of 3.06. *Fusarium solani* has been reported as highly pathogenic on date palms by Alwashi et al. [15] in a plantlet assay in UAE, and by Saleh et al. [3] in a detached leaflets assay in Saudi Arabia. On the contrary, Nishad and Ahmed [16], testing in Qatar the pathogenicity of *F. solani* on a detached leaf assay, reported that the strain tested was little virulent. In all papers mentioned above, the identification of *F. solani* was carried out by using different molecular markers. The FSSC species members cause foot and root rot of numerous crops, and the complex includes over 80 phylogenetically distinct species, including saprophytes, plant, animal, and human pathogens [33,34]. Since the previous reports on *F. solani* from date palms, no details were provided on which species member of the FSSC was tested for pathogenicity. The dramatic difference of *F. solani* pathogenicity, reported by several authors, could be related to different phylogenetic species of the complex tested in each paper. Our data confirm that members of FFSC can be highly pathogenic. Therefore, it is worrisome that this species can also occur on wilted date palm plants in Tunisia, as this species, together with *F. proliferatum*, is considered an increasing pathogen in palm date growing countries [3,15]. The three species of FIESC identified in this study and *F. brachygibbosum*, a species member of FSAMSC, proved to be highly pathogenic on date palms in our assay. Our data are in contrast with previous reports, since both FIESC members and *F. brachygibbosum* were evaluated as weakly or moderately pathogenic species [13,16]. In particular, both *F. equiseti*, a member of FIESC, and *F. brachygibbosum*, proved to be moderately pathogen in Qatar, in a date palm detached leaf assay [16], while *F. brachygibbosum* was reported to be a weak pathogen of roots in Oman [13]. It is important to underline that recent new taxonomic analyses of both FIESC and *F. brachygibbosum* have generated new phylogenetic species in FIESC [35] and split *F. brachygibbosum* in 12 phylogenetic new species grouped in *F. brachygibbosum* clade within FSAMSC [36]. Therefore, the different pathogenicity on date palm shown by FIESC members and *F. brachygibbosum* in different assays, reported in different papers, can be due to different phylogenetic species tested. 

### 3.3. Mycotoxin Profile

In addition to their pathogenicity ability that causes loss of production, all *Fusarium* species identified in this survey are also reason of high concern since they can produce a wide range of mycotoxins [19]. Their occurrence on date palm plants indicates that a contamination of the fruit at the harvest in the field and its by-products in postharvest can occur. Since we proved a high presence of toxigenic *Fusarium* species in some organs of the date palm plants (roots and leaves), to monitor environmental conditions in the field suitable for mycotoxin production in planta by the *Fusarium* species is important to manage and avoid an eventual, although unlike, final contamination of fruits. Our molecular and phylogenetic results showed that most of the identified *Fusarium* strains belong to *F. proliferatum*, a species member of the FFSC, while only a very small set of strains belonged to other complexes (FSAMSC, FIESC, FSSC). A correct identification of *Fusarium* species is a key aspect, since many species have a specific own mycotoxin profile. Therefore, accurate risk assessment is strongly linked to the use of advanced diagnostic tools. *Fusarium proliferatum* is characterized by a distinct mycotoxin profile. The in vitro mycotoxin production by the strains tested was analyzed with respect to the main mycotoxins associated to this species: FBs, MON, BEA, and ENNs. The data showed that two different groups of strains occurred with respect to FBs production, within *F. proliferatum*. A group of six strains that were isolated from three different localities (Degueche, Hezoue, Mides) did not produce any of the three FBs analyzed, while a group of 41 strains produced all FBs. These data could be comforting since a ratio of non-producing strains reduces the risk related to the occurrence of such important FBs producing species in the date palm. *Fusarium proliferatum* is a polyphagus species able to colonize a very wide range of crops [37], with a mixed population including FBs producers and FBs-not producers. We have reported that among *F. proliferatum* strains isolated from some crops (e.g., fig, wheat), strains unable to produce FBs in laboratory conditions could co-exist with FB-producing strains, even though they keep the whole biosynthetic FB gene cluster, with the same syntheny observed in FBs producers [38]. Similarly, in *Aspergillus* genus, we identified in two FB_2_ producing species, *Aspergillus niger* and *Aspergillus welwitschiae* [39] both chemotypes: FB_2_ producing and non-producing strains [40]. Therefore, the cause of lack of FB production in some of *F. proliferatum* strains isolated from date palm remains to be evaluated, to determine whether it is a conserved trait, due to possible mutations of one or more genes in the FB biosynthetic gene cluster. Indeed, the phylogenetically closest species to *F. proliferatum*, *Fusarium fujikuroi*, considered a sibling species of *F. proliferatum* [41], has been shown to include two phylogenetic groups where all strains of the so-called G-group lacked to produce FBs [42]. Associated to this trait, the authors proved the occurrence of three mutations that involved three different *FUM*-cluster genes essential for FBs production, where each mutation alone could account for the lack of FBs production [43,44]. If these mutations could occur also in the *F. proliferatum* strains lacking the FBs production capability is a hypothesis under evaluation. On the other hand, the high occurrence of *F. proliferatum* on date palm in Tunisia and the high incidence of highly FBs producing strains is worrisome, since both FB_1_ and FB_2_ are considered by the IARC group 2B, meaning potentially carcinogenic. The reports on the occurrence of this species on date fruit bunch in Iran and Israel, where *F. proliferatum* strains caused fruit drying and dropping [9,10], confirm it the concern for human health related to this species occurrence on date palm. Moreover, all strains analyzed produced BEA, while a significant group of them produced also ENNs A and B. Altogether, these compounds are cyclic hexadepsipeptides and have been reported to be not only toxic to humans and animals but also to possess phytotoxic activity [45,46]. Beauvericin production has been reported in several species of the genus *Fusarium* including different *formae speciales* of *F. oxysporum* [47,48,49]. Beauvericin can act as virulence factor for phytopathogenic *Fusarium* species, as reported by Lopez-Berges et al. [50] that proved that BEA deficient mutants of *F. oxysporum* were attenuated in virulence on tomato plants. Moreover, BEA was shown to be an intracellular phytotoxin and an inhibitor of respiration in young maize leaves [51], while in tomato BEA acts as an intermediate of cell death signaling, by causing an imbalance in the ascorbate system [45]. On the other hand, also ENNs were proved to be phytotoxic to plants. Mutants of the cereal pathogen *F. avenaceum* lacking enniatin synthetase exhibited significantly reduced virulence in an infection assay on potato tubers [52]. Therefore, the large production of cyclic hexadepsipeptides by the *F. proliferatum* isolated from date palm shows that this species has a high potential of using these compounds to increase its virulence to the plants. The ability of producing BEA and, more rarely, ENNs, was proved also for the strains of the FIESC species, especially *F. clavum*, for *F. solani*, and, at a low level for *F. brachygibbosum*. This shows that all these species can have a different potential to increase their virulence on plants by using cyclic hexadepsipeptides. We also analyzed the ability of all species to produce MON, a mycotoxin that plays a role in various animal mycotoxicoses, especially in poultry fed contaminated maize [53]. More recently, however, EFSA considered MON a low risk for humans and animals [54]. On the other hand, MON was often reported to have phytotoxic effects, as it can inhibit plant growth by reducing the efficiency of photosynthetic pigments, and may inhibit leaf development and can also reduce the mass of wheat plantlets [55]. Moniliformin was produced in very low concentrations by only some strains of *F. proliferatum*, but at high levels by both strains of *F. solani*. Interestingly, the latter species, the most pathogenic in our assay and reported as highly pathogenic on date palm in previous reports [3,15], was able to produce in vitro high amounts of MON, BEA, and some of the tested ENNs (ENNA1 and ENNH). These are all metabolites that could increase the virulence of *F. solani* against date palm under field conditions. Finally, the production of ZEN and trichothecenes was analyzed for the strains of FIESC, FSAMSC, and FSSC. No strain produced in vitro ZEN, while, among trichothecenes, only T-2 and HT-2 were produced by three strains of *F. brachygibbosum* along with DAS and NEO in traces. To the best of our knowledge, this is the first report demonstrating that *F. brachygibbosum* is capable of producing these mycotoxins, which are considered to be the most toxic *Fusarium* mycotoxins for humans and animals [19]. Previous studies reported the production of type B trichothecenes fusarenon X and 4,15diacetoxy-nivalenol by *F. brachygibbosum* isolated from legume pastures in Australia [56] and soybean roots in Ethiopia [57]. On the other hand, more recently, Laraba et al. [36] reported that strains of *F. brachygibbosum* isolated from Virgin jungle soil in Thailand and pearl millet in Niger could produce the type A trichothecenes diacetoxyscirpenol and neosolaniol. Our report is consistent with Laraba et al. [36], since DAS and NEO are well-known precursors of T-2 and HT-2 type A trichothecenes. Only a single species of *Fusarium* has been reported to be able to produce both types of trichothecenes, *Fusarium poae* [19], therefore further studies are in progress, aimed to analyze the trichothecene gene cluster of *F. brachygibbosum* and to obtain more accurate information on the potential mycotoxin profile of this species. The occurrence of such toxigenic species in date palms is reason of further concern, also since its occurrence has been often detected in our survey, in combination with the FB producing *F. proliferatum*, and therefore the risk of multiple mycotoxins contamination of fruits increases the risk for human health. 

## 4. Conclusions

To the best of our knowledge, currently in Tunisia, there is a lack of information on mycotoxin contamination of date fruits and related by-products. For the first time, we reported the occurrence of several *Fusarium* species on date palms grown in Tunisia. However, we could confirm that, although we detected a high incidence of *Fusarium* species on date palms, the quarantine pathogen *F. oxysporum* f. sp. *albedinis* is absent in all the considered samples. Among the *Fusarium* species identified, we reported a frequent occurrence of *F. proliferatum* as main species associated to date palm, confirming previous reports from Asia and Europe. This is a reason of serious concern, since this species produces the mycotoxins FBs, associated to a wide number of mycotoxicoses. We also provided new information on *F. brachygibbosum*, which was shown in our report for the first time to be able to produce T-2 and HT-2 toxins and on the additional potential contamination by cyclic hexadepsipeptides BEA and ENNs. Our data suggest the need of further investigations (i) for a better understanding of the possibility that *Fusarium* species occurring on date palm can produce mycotoxins in planta, (ii) for defining which environmental conditions would influence such production, useful for establishing proper management protocols to be adopted to minimize production loss, and (iii) to investigate whether *Fusarium* mycotoxins can actually occur in normal-looking fruits. Finally, our phylogenetic studies confirm that, also on date palm, the detection of multiple *Fusarium* species brings us, by using Monica Evans words, “*into the invisible, indispensable world of microbial biodiversity*”.

## 5. Materials and Methods

### 5.1. Origin of the Samples and Fungal Isolation

During 2017–2018, date-palm leaflets and roots showing fungal disease symptoms were randomly collected from 7 different Tunisian oases: Hazoua (33°43′49.81′′ N, 7°35′23.44′′ E), IBN Chabbat (33°55′53.34′′ N, 8° 4′57.07′′ E), Mides (34°24′31.97′′ N, 7°55′7.56′′ E), Tozeur (33°54′39.71′′ N, 8°8′29.90′′ E), Nafta (33°52′34.09′′ N, 7°52′44.55′′ E), and Degueche (33°59′32.39′′ N, 8°14′19.26′′ E), and El-Hamma (33°59′51.70′′ N, 8°9′36.31′′ E), located in Tozeur governorate.

After a surface-disinfection with 2% sodium hypochlorite solution for 2 min and two washings with distilled sterilized water for 1 min, leaf and roots portions, taken from the margin of the symptomatic tissues, were cut into small pieces (5 mm in diameter) with a sterilized scalpel and transferred on Potato Dextrose Agar (PDA) amended with 100 mg L^−1^ of streptomycin sulphate salt and 50 mg L^−1^ of neomycin. Petri dishes were incubated at 25 ± 1 °C for 7 days under an alternating light/darkness cycle of 12 h photoperiod. After incubation, fungal colonies originated from plant tissues and morphologically identified as *Fusarium* species, were transferred on new PDA plates and then purified by using the single spore isolation technique. All representative 63 monoconidial *Fusarium* strains (Table 3), 54 isolated from leaves, and 9 isolated from roots, were stored at −80 °C in 10% glycerol, as suspensions of conidia and mycelium, for further analyses.

### 5.2. DNA Extraction and Molecular Analyses

Sixty-three *Fusarium* monoconidial strains were firstly cultured on PDA medium, and after 3 days of incubation, 5 mycelial plugs from the margins of actively growing colonies were transferred on cellophane disks overlaid on PDA plates. Pure cultures were further incubated at 25 °C for 3 days and then mycelium of each strain was collected by scraping and lyophilized. DNAs of single pure cultures was isolated and purified from powdered lyophilized mycelium (10–15 mg) using the “Wizard Magnetic DNA Purification System for Food” kit (Promega Corporation, Madison, WI, USA), according to the manufacturer’s protocol. Integrity of DNA was checked by electrophoretic analysis on 0.8% agarose gel and by comparison with a standard DNA (1 kb DNA Ladder, Fermentas GmbH, St. Leon-Rot, Germany); quantity was evaluated by Thermo-Scientific Nanodrop (LabX, Midland, ON, Canada). Translation elongation factor, *CAL1* and *RPB2* were selected among the most informative housekeeping genes for the molecular characterization and for building phylogenetic relationships at inter- and intra-specific levels of *Fusarium* strains, using a multi-locus sequence approach. Polymerase chain reaction (PCR) reactions were set up using the following specific primer pairs and related amplification protocols: EF1/EF2 [58], CL1/CL2 [59], 5F/7CR [60]. PCR mixture (15 μL) containing approximately 15–20 ng of DNA template, 1.5 μL (10X) PCR solution buffer, 0.45 μL each primer (10 mM), 1.2 μL dNTPs (2.5 mM), and 0.125 μL of Hot Start Taq DNA Polymerase (1 U/μL; Fisher Molecular Biology, Trevose, PA, USA). For each reaction, a not template control was included to ascertain the absence of contamination. The PCR products, stained with GelRed® (GelRed® Nucleic Acid Gel Stain, 10,000X, Biotium Inc., Fremont, CA, USA), were visualized with UV after electrophoretic separation in 1X TAE buffer, on 1.5% agarose gel and sized by comparison with 100 bp DNA Ladder (Invitrogen, Thermo Fisher Scientific, Carlsbad, CA, USA). For DNA sequencing, PCR residual primers were removed using the enzymatic mixture EXO/FastAP (ExonucleaseI, FastAP thermosensitive alkaline phosphatase, Thermo Scientific, Vilnius, Lithuania) and PCR ampliocs were labelled using the BigDye Terminator v3.1 Cycle Sequencing Ready Reaction Kit (Applied Biosystems, Foster City, CA, USA), according to the manufacturer’s recommendations. Reaction of both strands were purified by filtration through Sephadex G-50 (5%) (Sigma-Aldrich, Saint Louis, MO, USA) and analyzed using “ABI PRISM 3730 Genetic Analyzer” (Applied Biosystems, Foster City, CA, USA). Partial FASTA sequences were analyzed and assembled using the BioNumerics v. 5.1 software (Applied Maths, Kortrijk, Belgium). DNA sequences of *Fusarium* species reference strains (Table 2) were downloaded through the National Center for Biotechnology Information (NCBI). All sequences of the 3 genes (*TEF1*, *CAL1*, *RPB2*) were aligned using the ClustalW algorithm [61] and the phylogenetic relationships were built using the maximum likelihood method with the MEGA software version 7 [62]. The bootstrap analysis [63] was conducted to determine the confidence of internal nodes using a heuristic search with 1000 replicates, removing gaps. 

### 5.3. Pathogenicity Assay

Pathogenicity assays were carried out by using 51 strains, representing all species identified in this study (Table S1). The strains were grown for 7–10 days at 25 °C on PDA under a light/darkness photoperiod of 12 h. For each strain, the inoculum was prepared by flooding the agar plate surface with 10 mL of sterile distilled water (SDW) and scraping with a spatula. Conidial suspensions were filtered through four layers of cheese cloth and were diluted with SDW for obtaining a final concentration of 10^5^ conidia mL^−1^ [64]. 

The pathogenicity tests were performed on healthy date palm plantlets of the most common Tunisian cultivar *Deglet Nour*. The plantlets used were regenerated from direct somatic embryogenesis, derived from shoot-tip explants. Therefore, they were acclimatized in greenhouse under controlled conditions, for 6-months. The plants were of 25–30 cm length, having 4–5 leaves, and have grown in large pots (12 cm diameter and 18 cm height filled with planting medium peat/perlite 2:1 (v/v)). Each plantlet was inoculated by injecting 2 mL of conidial suspension into the plantlet crown area, using a sterile hypodermic syringe. Three plantlets per treatment were considered, and each experiment was performed in triplicate. Date palm plantlets injected only with SDW were used as negative control. To favor fungal development, all plantlets were watered and covered with plastic bags for 24 h, in order to ascertain high moisture content. Plantlets were growth in greenhouse at 25 ± 2 °C, and 80% RH, 12 h light/darkness photoperiod. After inoculation, disease symptoms were assessed weekly for 3 months.

Pathogenicity was evaluated by assessing the degree of disease symptoms in the plantlets, according with Alwahshi et al. [15], partially adapted. Each plantlet was rated on a scale of 0–4, where:

0 indicated a healthy plantlet with a well-developed foliar apparatus that exhibited negligible symptoms; 

1 indicated plants with up to 25% of disease symptoms in crown area, exhibiting necrotic lesions from 5 to 10 mm and beginning of whitening; 

2 indicated plants with symptoms from 25% to 50% of disease symptoms, exhibiting necrosis and wilting lesions from 11 to 30 mm; 

3 indicated plants with symptoms from 50 to 75% exhibiting extensive necrotic lesions (30–70 mm); 

4 indicated plants with symptoms more than 75% of crown area, including also necrosis and whitening of leaves and root rot symptoms. 

To confirm Koch’s postulates, pieces of leaves, where symptoms of the disease appeared, were sterilized on the surface and re-isolated. Plates were incubated at 25 ± 2 °C and subsequent growth was recorded.

Disease severity index (DSI) was calculated using the Townsend–Heuberger formula [65]: DSI = Σ [(n × c) ⁄ (V × N)] × 100, where n is number of palm plantlets per class, c is the numerical value of each class, V is the highest class value, and N is the total number of plantlets. Data of pathogenicity assays were subjected to analysis of variance (ANOVA) and Duncan’s multiple range test, by using Statistical Package for the Social Sciences (SPSS) v. 27.0 software, with significance level (P) of 0.05. 

### 5.4. Mycotoxin Analyses

#### 5.4.1. Sample Preparation

Sixty *Fusarium* strains were analyzed for the in vitro mycotoxins production (Table 2). In detail, 47 *F. proliferatum* strains were tested for the capability to produce MON, BEA, FBs (FB_1_, FB_2_, FB_3_), and ENNs (EnnA_1_, EnnB, EnnB_1_, EnnH); 6 *F. brachygibbosum* strains, 5 *Fusarium* strains of FIESC, and 2 *F. solani* strains were tested for the production of MON, BEA, FBs, ENNs, ZEA, and trichothecenes.

Each strain was grown on plates containing PDA and then mycelial plugs of these cultures were used to inoculate 30 g of autoclaved rice, previously kept to 45% of moisture for one night. Cultures were incubated at 25 °C in darkness for 3 weeks, then dried at 65 °C for 24 h and ground to a fine powder and used for chemical analyses. Not inoculated rice, treated in the same way, was used as negative control. Samples were prepared according to Malachová et al. [66] procedure. Briefly, 0.5 g of ground cereal was stirred for 90 min at 200 strokes/min on a shaker with 2 mL of acetonitrile/water (80/20, v/v) mixture acidified with 0.2% of formic acid and then centrifuged for 10 min at 14,000 rpm. A total of 1 µL of supernatant was injected into LC-MS.

#### 5.4.2. Chemicals and Reagents

Mycotoxin standard solutions (in acetonitrile) were obtained from Romer Labs (Tulln, Austria).

LC-MS grade methanol and acetonitrile were purchased from Scharlab Italia srl (Milan, Italy); bidistilled water was obtained using Milli-Q System (Millipore, Bedford, MA, USA). MS-grade ammonium acetate, acetic acid, and formic acid from Fisher Chemical (Thermo Fisher Scientific Inc., San Jose, CA, USA) were also used.

#### 5.4.3. Targeted UHPLC-MS/MS Analysis of MON

UHPLC Dionex Ultimate 3000 separation system coupled to a triple quadrupole mass spectrometer (TSQ Vantage; Thermo Fisher Scientific Inc., San Jose, CA, USA) equipped with an electrospray source (ESI) was employed.

For the chromatographic separation, XBridge Amide BEH column (Waters, Wilmslow, UK) with 2.10 × 100 mm and a particle size of 2.6 µm heated to 40 °C was used. A total of 2 μL of sample extract was injected into the system; the flow rate was 0.4 mL/min.

Gradient elution was performed by using acetonitrile (eluent A), water acidified with 0.2% formic acid (eluent B) and ammonium formate 20 mM 1% formic acid (eluent C). The elution gradient started from 0% of B, 5% of C and 95% of A and, after an initial isocratic step of 2 min, B increased at 60% in 3 min and this composition is kept for 2.5 min. At 8 min, the initial conditions were restored and kept for 5 min. The total run time was 13 min.

Moniliformin was monitored in negative ionization mode; spray voltage 3500 V, capillary temperature at 270 °C, vaporizer temperature was kept at 200 °C, sheath gas flow was set at 50 units, and the auxiliary gas flow at 5 units. S-Lens RF amplitude value and collision energies (CE) were optimized during infusion of analyte standard solutions (1000 ng mL^−1^, in methanol) employing an automatic function of the Xcalibur software (Thermo Fisher Scientific, San Jose, CA, USA). Detection was performed using multiple reaction monitoring (MRM) mode. The following optimized transitions were used for the quantification: m/z 97→41 (CE 16 eV); m/z 97→80 (CE 23 eV); m/z 97→63 (CE 45 eV). Quantification of target analytes was performed using matrix-matched calibration standards (range 0.001–2 ppm). 

#### 5.4.4. UHPLC-TWIMS-QTOF Screening of Mycotoxins

ACQUITY I-Class UPLC separation system coupled to a VION IMS QTOF mass spectrometer (Waters, Wilmslow, UK) equipped with an electrospray ionization (ESI) interface was employed for mycotoxins screening. Samples were injected (1 µL) and chromatographically separated using a reversed-phase C18 BEH ACQUITY column 2.1 × 100 mm, 1.7 µm particle size (Waters, Milford, MA, USA). A gradient profile was applied using water 1 mM ammonium acetate (eluent A) and methanol (eluent B) both acidified with 0.5% acetic acid as mobile phases. Initial conditions were set at 5% B, after 0.7 min of isocratic step, a linear change to 50% B in 5.8 min. Moreover, 100% B was achieved in 3 min and holding for 3 min to allow for column washing before returning to initial conditions. Column recondition was achieved over 1.5 min, providing a total run time of 14 min. The column was maintained at 40 °C and a flow rate of 0.4 mL/min used. 

Mass spectrometry data were collected in positive electrospray mode over the mass range of m/z 50−1000. Source settings were maintained using a capillary voltage, 1.0 kV; source temperature, 150 °C; desolvation temperature, 600 °C and desolvation gas flow, 1000 L/h. The TOF analyzer was operated in sensitivity mode and data acquired using HDMSE, which is a data independent approach (DIA) coupled with ion mobility. The optimized ion mobility settings included: nitrogen flow rate, 90 mL/min (3.2 mbar); wave velocity 650 m/s and wave height, 40 V. Device within the Vion was calibrated using the Major Mix IMS calibration kit (Waters, Wilmslow, UK) to allow for CCS values to be determined in nitrogen. The calibration covered the CCS range from 130–306 Å2. The TOF was also calibrated prior to data acquisition and covered the mass range from 151 Da to 1013 Da. TOF and CCS calibrations were performed for both positive and negative ion mode. Data acquisition was conducted using UNIFI 1.8 (Waters, Wilmslow, UK).

Mycotoxins identification was performed by comparison of rt, fragmentation pattern and collision cross-sections with the standard collect in our UNIFI library, created by running a mix of standards with the same analytical method. Quantification of target analytes was performed using calibration standards (range 0.1–2 ppm). 

## Figures and Tables

**Table 1:** *Fusarium* reference strains used in this study for phylogenetic analysis.

*Fusarium* species	Strain	GenBank Assembly Accession			Database *	References/Submitter
		*CAL1*	*RPB2*	*TEF1*		
*F. brachygibbosum*	NRRL 34033	GQ505388	GQ505482	GQ505418	NCBI	[24]
*F. caatingaense*	NRRL 66470	GCA_013624355	GCA_013624355	GCA_013624355	NCBI	USDA
*F. clavum*	NRRL_66337	GCA_004367155	GCA_004367155	GCA_004367155	NCBI	USDA
*F. culmorum*	FcUK99	GCA_900074845	GCA_900074845	GCA_900074845	NCBI	[25]
*F. equiseti*	D25-1	GCA_003313175	GCA_003313175	GCA_003313175	NCBI	[26]
*F. fujikuroi*	IMI 58289	GCA_900079805	GCA_900079805	GCA_900079805	NCBI	HMGU-IBIS
*F. graminearum*	PH-1	GCA_900044135	GCA_900044135	GCA_900044135	NCBI	[27]
*F. langsethiae*	Fl201059	GCA_001292635	GCA_001292635	GCA_001292635	NCBI	[28]
*F. proliferatum*	ET1	GCA_900029915	GCA_900029915	GCA_900029915	NCBI	HMGU-IBIS
*F. solani*	PUF007	HQ412317	HQ423201	HQ165838	NCBI	[29]
*F. sporotrichioides*	NRRL3299	GCA_003012315	GCA_003012315	GCA_003012315	NCBI	[30]
*F. verticillioides*	7600	GCA_000149555	GCA_000149555	GCA_000149555	NCBI	[31]

* NCBI = National Center for Biotechnology Information; USDA: US Department of Agriculture, Agriculture Research Service; HMGU-IBIS: Helmholtz Zentrum München—German Research Center for Environmental Health—Institute of Bioinformatics and Systems Biology.

**Table 2:** Mycotoxin production by *Fusarium* strains isolated from date palm in Tunisia (mg kg^−1^).

Strain	Fumonisins			MON	BEA	Enniatins				Trichothecenes		
	FB1	FB2	FB3			ENN A1	ENN B	ENN B1	ENN H	ZEN	HT2	T2
*Fusarium proliferatum*												
18583	240.2	31.8	413.7	2.4	17.7	<LOD				-	-	-
18584	22.1	7.1	803.1	21.7	75.9	<LOD				-	-	-
18585	133.9	362.3	18.7	<LOD	80.5	<LOD				-	-	-
18586	168.5	334.1	11.7	<LOD	52.2	<LOD				-	-	-
18587	1382.2	626.8	299.1	<LOD	39	<LOD				-	-	-
18588	32.1	14.03	287.9	<LOD	422.7	<LOD				-	-	-
18589	19.9	5.6	2.4	41.4	0.3	<LOD				-	-	-
18590	57.6	12	99.5	<LOD	0.8	<LOD				-	-	-
18591	45.3	7	65.2	<LOD	1.3	<LOD				-	-	-
18592	48	125.8	591.6	32.2	42.3	<LOD				-	-	-
18593	917.4	425.7	49.6	1.9	47	<LOD				-	-	-
18594	120.3	47.8	15	<LOD	1.8	<LOD				-	-	-
18595	461.1	121.6	544.2	17.2	29	<LOD				-	-	-
18596	719	140.2	35.2	<LOD	32.5	<LOD				-	-	-
18597	1472.3	808.9	290.8	<LOD	16	<LOD				-	-	-
18598	4.1	2.2	0.6	<LOD	0.1	<LOD				-	-	-
18600	1116.2	190.1	<LOD	<LOD	46	<LOD				-	-	-
18601	416.5	94.4	81	<LOD	7.2	<LOD				-	-	-
18602	8.7	6.2	2.2	<LOD	8.6	<LOD	0.01	<LOD	<LOD	-	-	-
18603	921.3	184.6	365.1	<LOD	97.7	<LOD				-	-	-
18604	763.3	104	273.7	<LOD	26.6	<LOD				-	-	-
18605	37	4.8	14.25	<LOD	20.8	<LOD				-	-	-
18606	1122.9	163.6	58	<LOD	77.5	<LOD				-	-	-
18607	55	36.7	60.7	1	84.6	112.40	48.6	33.7	51.1	-	-	-
18608	0.9	0.3	<LOD	<LOD	6	<LOD				-	-	-
18609	<LOD	<LOD	<LOD	<LOD	8.2	<LOD				-	-	-
18610	1649.2	5.9	<LOD	<LOD	40.5	<LOD				-	-	-
18611	1090.4	69.9	507	<LOD	260.2	<LOD				-	-	-
18612	1086.8	44.7	149.9	<LOD	336.6	<LOD				-	-	-
18613	2213.6	373.1	529.9	<LOD	420.6	<LOD				-	-	-
18614	4.2	1.8	1.4	<LOD	17.2	<LOD				-	-	-
18615	1.1	0.4	<LOD	<LOD	0.04	<LOD	0.1	<LOD	1.1	-	-	-
18616	0.8	0.3	<LOD	<LOD	12.5	<LOD				-	-	-
18617	<LOD	<LOD	<LOD	<LOD	10.4	<LOD				-	-	-
18618	<LOD	<LOD	<LOD	<LOD	210.5	<LOD				-	-	-
18619	<LOD	<LOD	<LOD	<LOD	211.7	<LOD				-	-	-
18620	<LOD	<LOD	<LOD	<LOD	33.4	<LOD				-	-	-
18621	<LOD	<LOD	<LOD	<LOD	437.4	<LOD				-	-	-
18622	1942.4	765.3	319.1	<LOD	290.6	<LOD				-	-	-
18623	319.2	44.2	49.8	<LOD	39.1	<LOD				-	-	-
18624	31	4.1	5	1	10	<LOD				-	-	-
18625	100.4	9.7	21.1	<LOD	46	<LOD				-	-	-
18626	9.8	1	2	<LOD	2.9	<LOD				-	-	-
18627	9	0.9	1.8	<LOD	3.9	<LOD				-	-	-
18628	11809.8	622.4	292.2	<LOD	8.6	<LOD				-	-	-
18629	1100.8	424.9	269.8	25	94.2	<LOD				-	-	-
18645	1222.2	234.6	425.3	<LOD	90.8	<LOD				-	-	-
*Fusarium brachygibbosum*												
18635	-	-	-	<LOD	1.2	<LOD	0.02	<LOD	1.9	<LOD	18.7	<LOD
18636	-	-	-	<LOD	0.4	<LOD				<LOD	<LOD	1.2
18637	-	-	-	<LOD	1.9	<LOD				<LOD		
18638	-	-	-	<LOD	1	<LOD				<LOD		
18639	-	-	-	<LOD	0.1	<LOD				<LOD		
18640	-	-	-	<LOD	0.6	<LOD				<LOD	<LOD	2.4
*Fusarium incarnatum-equiseti* species complex												
18630 *F. incarnatum*	-	-	-	<LOD	3.2	<LOD				<LOD		
18631	-	-	-	<LOD	0.5	<LOD				<LOD		
18632	-	-	-	<LOD	0.3	<LOD				<LOD		
18633	-	-	-	<LOD	0.9	<LOD				<LOD		
*F. caatingaense*												
18634 *F. clavum*	-	-	-	<LOD	423.1	<LOD				<LOD		
*Fusarium solani*												
18643	-	-	-	50.1	21.6	82.5	<LOD	<LOD	285	<LOD		
18644	-	-	-	62.2	25.2	95.1	8.2	<LOD	224.1			

**Table 3:** List of *Fusarium* strains considered in this study.

Strain (ITEM *)	*Fusarium* species	Part of Plant	Origin	Years of Sampling
18618	*F. proliferatum*	Leaflets	Degueche	2017
18619	*F. proliferatum*	Leaflets	Degueche	2017
18583	*F. proliferatum*	Leaflets	El-Hamma	2017
18584	*F. proliferatum*	Leaflets	El-Hamma	2017
18592	*F. proliferatum*	Leaflets	El-Hamma	2017
18595	*F. proliferatum*	Leaflets	El-Hamma	2017
18585	*F. proliferatum*	Leaflets	Hezoua	2017
18586	*F. proliferatum*	Leaflets	Hezoua	2017
18590	*F. proliferatum*	Leaflets	Hezoua	2017
18591	*F. proliferatum*	Leaflets	Hezoua	2017
18605	*F. proliferatum*	Leaflets	Hezoua	2017
18611	*F. proliferatum*	Leaflets	IBN Chabbat	2017
18612	*F. proliferatum*	Leaflets	IBN Chabbat	2017
18613	*F. proliferatum*	Leaflets	IBN Chabbat	2017
18589	*F. proliferatum*	Leaflets	Mides	2017
18624	*F. proliferatum*	Leaflets	Mides	2017
18627	*F. proliferatum*	Leaflets	Mides	2017
18606	*F. proliferatum*	Leaflets	Tozeur	2017
18607	*F. proliferatum*	Leaflets	Tozeur	2017
18610	*F. proliferatum*	Leaflets	Tozeur	2017
18623	*F. proliferatum*	Leaflets	Tozeur	2017
18602	*F. proliferatum*	Roots	Mides	2017
18616	*F. proliferatum*	Roots	Mides	2017
18636	*F. brachygibbosum*	Leaflets	IBN Chabbat	2017
18639	*F. brachygibbosum*	Leaflets	IBN Chabbat	2017
18640	*F. brachygibbosum*	Leaflets	IBN Chabbat	2017
18635	*F. brachygibbosum*	Leaflets	Mides	2017
18599	*F. clavum*	Leaflets	Mides	2017
18631	*F. caatingaense*	Leaflets	Tozeur	2017
18632	*F. caatingaense*	Leaflets	Tozeur	2017
18633	*F. caatingaense*	Roots	Tozeur	2017
18596	*F. proliferatum*	Leaflets	Degueche	2018
18621	*F. proliferatum*	Leaflets	Degueche	2018
18594	*F. proliferatum*	Leaflets	Hezoua	2018
18617	*F. proliferatum*	Leaflets	Hezoua	2018
18620	*F. proliferatum*	Leaflets	Hezoua	2018
18593	*F. proliferatum*	Leaflets	IBN Chabbat	2018
18597	*F. proliferatum*	Leaflets	IBN Chabbat	2018
18600	*F. proliferatum*	Leaflets	IBN Chabbat	2018
18601	*F. proliferatum*	Leaflets	IBN Chabbat	2018
18603	*F. proliferatum*	Leaflets	IBN Chabbat	2018
18622	*F. proliferatum*	Leaflets	IBN Chabbat	2018
18615	*F. proliferatum*	Leaflets	Mides	2018
18626	*F. proliferatum*	Leaflets	Mides	2018
18645	*F. proliferatum*	Leaflets	Mides	2018
18608	*F. proliferatum*	Leaflets	Mides	2018
18598	*F. proliferatum*	Leaflets	Mides	2018
18609	*F. proliferatum*	Leaflets	Mides	2018
18614	*F. proliferatum*	Leaflets	Mides	2018
18604	*F. proliferatum*	Leaflets	Nafta	2018
18628	*F. proliferatum*	Leaflets	Nafta	2018
18629	*F. proliferatum*	Leaflets	Nafta	2018
18625	*F. proliferatum*	Leaflets	Tozeur	2018
18588	*F. proliferatum*	Roots	IBN Chabbat	2018
18587	*F. proliferatum*	Roots	IBN Chabbat	2018
18641	*F. brachygibbosum*	Leaflets	El-Hamma	2018
18637	*F. brachygibbosum*	Leaflets	Mides	2018
18638	*F. brachygibbosum*	Roots	Hezoua	2018
18642	*F. brachygibbosum*	Roots	El-Hamma	2018
18634	*F. clavum*	Leaflets	IBN Chabbat	2018
18630	*F. incarnatum*	Roots	Mides	2018
18643	*F. solani*	Roots	IBN Chabbat	2018
18644	*F. solani*	Leaflets	Mides	2018

* = ITEM, Agri-Food Toxigenic Fungi Culture Collection, ISPA, Bari. http://www.ispa.cnr.it/Collection (accessed on 3 May 2021).

## Data Availability

The data presented in this study are available in Rabaaoui, A.; Dall’Asta, C.; Righetti, L.; Susca, A.; Logrieco, A.F.; Namsi, A.; Gdoura, R.; Werbrouck, S.P.O.; Moretti, A.; Masiello, M. Phylogeny and Mycotoxin Profile of Pathogenic *Fusarium* species Isolated from Sudden Decline Syndrome and Leaf Wilt Symptoms, on Date Palm (*Phoenix dactylifera*), in Tunisia. Toxins 2021, 13, 463. doi: 10.3390/toxins13070463.

## Supplementary Materials

The following are available online at https://www.mdpi.com/article/10.3390/toxins13070463/s1, Table S1—Pathogenicity of 51 *Fusarium* strains selected for pathogenicity assay. For each experiment, 3 replications consisting of 3 plants per replicate were considered. Data were analyzed using one-way ANOVA, followed by means separation using Duncan test at *p* ≤ 0.05.
