# Supplementary material for: Phylogeny and Mycotoxin Profile of Pathogenic Fusarium Species Isolated from Sudden Decline Syndrome and Leaf Wilt Symptoms on Date Palms (Phoenix dactylifera) in Tunisia
Source: Toxins (Basel). 2021 Jun 30;13(7):463. doi: 10.3390/toxins13070463 (PMC8310299; doi:10.3390/toxins13070463)
Supplement: Supplementary file 1 [file toxins-13-00463-s001.zip › toxins-1269074-supplementary.pdf]

Supplementary Materials: Phylogeny and Mycotoxin Profile of Pathogenic *Fusarium* species Isolated from Sudden Decline Syndrome and Leaf Wilt Symptoms, on Date Palm (*Phoenix dactylifera*), in Tunisia

Amal Rabaaoui, Chiara Dall'Asta, Laura Righetti, Antonia Susca, Antonio F. Logrieco, Ahmed Namsi, Radhouane Gdoura, Stefaan P. O. Werbrouck, Antonio Moretti and Mario Masiello

Table S1. Pathogenicity of 51 *Fusarium* strains selected for pathogenicity assay. For each experiment, 3 replications consisting of 3 plants per replicate were considered. Data were analyzed using one-way ANOVA, followed by means separation using Duncan test at $p \leq 0.05$.

Strain (ITEM)	*Fusarium* species	Part of Plant	Origin	DSI*
**18583**	*F. proliferatum*	Leaves	El-Hamma	38.9^{a**}
**18584**	*F. proliferatum*	Leaves	El-Hamma	50^{abcd}
**18595**	*F. proliferatum*	Leaves	El-Hamma	50^{abcd}
**18592**	*F. proliferatum*	Leaves	El-Hamma	47.2^{abcd}
		**Mean value**		**46.5**
**18585**	*F. proliferatum*	Leaves	Hezoua	55.6^{abcd}
**18586**	*F. proliferatum*	Leaves	Hezoua	50^{abcd}
**18590**	*F. proliferatum*	Leaves	Hezoua	44.4^{abcd}
**18591**	*F. proliferatum*	Leaves	Hezoua	41.7^{ab}
**18594**	*F. proliferatum*	Leaves	Hezoua	44.4^{abc}
**18605**	*F. proliferatum*	Leaves	Hezoua	63.9^{abcd}
**18620**	*F. proliferatum*	Leaves	Hezoua	63.9^{abcd}
		**Mean value**		**52**
**18593**	*F. proliferatum*	Leaves	IBN Chabbat	72.2^{abcd}
**18601**	*F. proliferatum*	Leaves	IBN Chabbat	63.9^{abcd}
**18612**	*F. proliferatum*	Leaves	IBN Chabbat	83.3^{d}
**18597**	*F. proliferatum*	Leaves	IBN Chabbat	69.4^{abcd}
**18600**	*F. proliferatum*	Leaves	IBN Chabbat	72.2^{abcd}
**18588**	*F. proliferatum*	Roots	IBN Chabbat	61.1^{abcd}
**18587**	*F. proliferatum*	Roots	IBN Chabbat	72.2^{abcd}
		**Mean value**		**70.6**
**18608**	*F. proliferatum*	Leaves	Mides	75^{bcd}
**18609**	*F. proliferatum*	Leaves	Mides	66.7^{abcd}
**18626**	*F. proliferatum*	Leaves	Mides	66.7^{abcd}
**18598**	*F. proliferatum*	Leaves	Mides	75^{bcd}
**18614**	*F. proliferatum*	Leaves	Mides	69.4^{abcd}
**18615**	*F. proliferatum*	Leaves	Mides	66.7^{abcd}
**18589**	*F. proliferatum*	Leaves	Mides	75^{bcd}
**18627**	*F. proliferatum*	Leaves	Mides	69.4^{cd}
		**Mean value**		**70.5**
**18607**	*F. proliferatum*	Leaves	Tozeur	72.2^{abcd}
**18623**	*F. proliferatum*	Leaves	Tozeur	75^{abcd}
**18625**	*F. proliferatum*	Leaves	Tozeur	80.6^{abcd}

**18610**	*F. proliferatum*	Leaves	Tozeur	61.1[abcd]
**18606**	*F. proliferatum*	Leaves	Tozeur	58.3[abcd]
		**Mean value**		**69.4**
**18596**	*F. proliferatum*	Leaves	Dgeuch	63.9[abcd]
**18621**	*F. proliferatum*	Leaves	Dgeuch	47.2[abcd]
**18619**	*F. proliferatum*	Leaves	Dgeuch	50[abcd]
		**Mean value**		**53.7**
**18604**	*F. proliferatum*	Leaves	Nafta	63.9[abcd]
**18628**	*F. proliferatum*	Leaves	Nafta	52.8[abcd]
**18629**	*F. proliferatum*	Leaves	Nafta	58.3[abcd]
		**Mean value**		**58.3**
			**MIN**	**38.9**
	Fusarium proliferatum		**MAX**	**83.3**
			**Mean value**	**61.9**
**18630**	*F. incarnatum*	Roots	Mides	88.9[a]
**18631**	*F. caatingaense*	Leaves	Tozeur	58.3[ab]
**18632**	*F. caatingaense*	Leaves	Tozeur	63.9[ab]
**18633**	*F. caatingaense*	Roots	Tozeur	72.2[b]
**18634**	*F. clavum*	Leaves	IBN Chabbat	80.6[ab]
			**MIN**	**58.3**
	**Fusarium Incarnatum Equiseti Species Complex**		**MAX**	**88.9**
			**Mean value**	**72.8**
**18635**	*F. brachygibbosum*	Leaves	Mides	80.6[b]
**18637**	*F. brachygibbosum*	Leaves	Mides	75[ab]
**18638**	*F. brachygibbosum*	Roots	Hezoua	47.2[ab]
**18639**	*F. brachygibbosum*	Leaves	IBN Chabbat	69.4[ab]
**18640**	*F. brachygibbosum*	Leaves	IBN Chabbat	63.9[ab]
**18641**	*F. brachygibbosum*	Leaves	El-Hamma	41.7[ab]
**18642**	*F. brachygibbosum*	Roots	El-Hamma	47.2[a]
			**MIN**	**41.7**
	Fusarim brachygibbosum		**MAX**	**80.6**
			**Mean value**	**60.7**
**18643**	*F. solani*	Roots	IBN Chabbat	72.2[a]
**18644**	*F. solani*	Leaves	Mides	80.6[a]
			**MIN**	**72.2**
	Fusarium solani		**MAX**	**80.6**
			**Mean value**	**76.4**

* DSI = Σ [(n × c) ⁄ (V × N)] × 100, where n is number of palm plantlets per class, c is the numerical value of each class, V is the highest class value, and N is the total number of plantlets. **= a, b, c, d: values followed by the same letter are not significantly different ($p < 0.05$) according to Duncan test.
